# A Positive Psychology Resource for Students? Evaluation of the Effectiveness of the 6 Minutes Diary in a Randomized Control Trial

**DOI:** 10.3389/fpsyg.2022.896741

**Published:** 2022-05-31

**Authors:** Timo Lorenz, Mona Algner, Benjamin Binder

**Affiliations:** Department of Psychology, Medical School Berlin (MSB), Berlin, Germany

**Keywords:** positive psychology, intervention, randomized control group design, resilience, stress, longitudinal design, well-being, self-efficacy

## Abstract

This study investigated the effects of the 6 Minutes Journal (6MT), a commercial diary combining several positive psychology interventions, including gratitude, goal-setting, and self-affirmation exercises, on several mental health outcome measures. In a randomized controlled trial, university students (*N* = 157) were randomly assigned to one of two groups: 6MT (*n* = 77) and a wait list control group (*n* = 80). Participants in the intervention group were instructed to follow the instructions of the 6MT for 4 weeks. Participants in both groups completed measures of perceived stress, positive and negative affect, self-efficacy and resilience at baseline, after 2 (t1), and 4 (t2) weeks. We used path-analyses with autoregressive and cross-lagged effects to test our hypotheses of the effects of the 6MT. Participants in the intervention group reported decreased levels of perceived stress and negative affect, as well as increased levels of resilience and self-efficacy compared to the control group. Positive affect was not statistically significantly influenced. The data showed a statistically significant increased levels of self-efficacy and resilience only after 4 weeks, suggesting that changing these constructs needs more time. The 6-minute diary does not appear to make individuals fundamentally more positive. However, the intervention may have a protective function against negative influences on well-being.

## Introduction

Stress continues to be a major health concern for university students (Çivitci, 2015; Garett et al., 2017). The unique challenges students face exposes them to a range of stressors (Acharya et al., 2018), resulting in an increasing number of students reporting moderate levels of stress-related mental health problems (Regehr et al., 2013). Not only is chronic stress negatively correlated with mental health, but studies show significant effects on student academic performance and a decrease in student success (American College Health Association, 2021).

These phenomena were the case before the outbreak of COVID-19 disease (Wang et al., 2020) and have increased since (Cao et al., 2020; Chi et al., 2020; Charles et al., 2021). Given the high demands and little resources regarding time and income, there is a need for fast and low-cost interventions as resources for this target group against stress and its impact on health in everyday student life, but increasingly so during crises.

The use of evidence-based positive interventions has been shown in previous studies to have desirable associations with relevant outcome measures, such as functional coping with stress (Layous et al., 2014), subjective well-being (Wood et al., 2009; Rash et al., 2011; Allan et al., 2013; Datu, 2013) or social relationships (Emmons and McCullough, 2003). Recent meta-analytic evidence suggests that positive interventions have at least small to medium-sized positive effects on well-being (Donaldson et al., 2021) and when used in workplace settings resulted in small to medium effects on both desirable (e.g., engagement, prosocial behavior) and undesirable outcomes (e.g., stress; Donaldson et al., 2019).

### The Current Study

In this study, we aim to investigate the effect of the 6-Minute diary on several outcomes in a student population. The 6-Minute diary is a commercial product that includes an introductory part consisting of information on the positive effects of positive psychology, habits, and self-reflection, as well as the actual daily diary section. The diary section includes a collection of various evidence-based positive psychology interventions. There are three short exercises per day in the morning and three exercises in the evening.

Apart from the daily interventions, the diary has sections for a monthly check-in, in which the users can reflect on several personal outcomes (e.g., mood, exercise, health, or finances). In the following section, these interventions are introduced and previous evidence and possible modes of action are explained.

According to a recent systematic review by Donaldson et al. (2021), the most promising positive psychology interventions consist of several components in order to be effective. These interventions offer the possibility to learn (i.e., develop knowledge and awareness), practice (i.e., behavioral skills easily implemented in daily life), reflect (i.e., sense-making and reinforcement after exercises), relate (i.e., increase engagement and accountability), and plan (i.e., goal setting and planning to ensure sustainability; Donaldson et al., 2021). The 6-Minute diary combines several of the mentioned components, such as monthly reflection, interventions and educational sections. The aim of our study is to take the previous results from positive psychology studies and test them in a commercial, i.e., a purchasable product on the market, and evaluate them in such a real life form. Furthermore, we want to test the possible effect of the 6 Minutes Diary on variables related to possible preventive effects on physical and mental health. The interventions included in the 6 Minutes Diary and their expected effects on the dependent variables are explained in the next sections.

#### Gratitude Intervention

One of the exercises in the 6-Minute Diary is a classic gratitude intervention in which one lists things or events for which one is grateful (Emmons and McCullough, 2003; Watkins et al., 2003; Seligman et al., 2005; Froh et al., 2008). To date, several studies have examined the benefits of gratitude for interpersonal relationships (Sun et al., 2014), social support (Wood et al., 2008), subjective well-being (Wood et al., 2009; Rash et al., 2011; Allan et al., 2013; Datu, 2013), strengthened social relationships (Emmons and McCullough, 2003), and improved physiological and cognitive functioning (McCraty et al., 2003). There are positive correlations between gratitude and life satisfaction, happiness, optimism, hope, and positive affect. Furthermore, there are negative correlations between gratitude and anxiety, depression, negative affect, and physical aggression (e.g., McCullough et al., 2002; Watkins et al., 2003). These results indicate the potency of gratitude as an amplifier for positive emotions and a buffer for negative emotions.

#### Goal Setting

Another exercise of the 6-Minute Diary is setting positive, personal goals. These goals reflect consciously formulated and personally meaningful objectives that guide perceptions, feelings, thoughts, and actions (Elliot et al., 2001; Wiese and Freund, 2005). Conscious goal setting is intended to direct attention and effort toward goal-relevant activities and away from goal-irrelevant activities (Locke et al., 1981).

There are negative correlations between goal-setting interventions and negative affect, and positive correlations with positive affect (Emmons and Diener, 1986), subjective well-being (Brunstein, 1993), and higher expectancy of success (Karakowsky and Mann, 2008). The mere existence of self-set goals seems to correlate with well-being as much as the actual achievement of the goals (Emmons and Diener, 1986). Furthermore, there is evidence for a positive influence of goal setting on perceived self-efficacy (e.g., Latham and Seijts, 1999).

#### Self-Affirmation

The principles of self-affirmation are also echoed through a 6-Minute diary exercise in which users write a positive phrase about themselves each morning. These phrases are designed to help identify and reinforce one’s core values (Steele, 1988; Sherman and Cohen, 2006).

Regular self-affirmations have been shown to reduce negative emotions (Nelson et al., 2014), moderate physiological responses to stressful situations (Creswell et al., 2005; Sherman et al., 2009), and increase self-efficacy expectations, positive affect (Howell, 2017), and academic achievement (Cohen et al., 2006, 2009).

#### Random Acts of Kindness Exercise

Another exercise in the 6-Minute diary draws on the principles of *random acts of kindness* (Jones, 1998). Individuals are asked to remember what good they have done for others that day. Being aware of one’s acts of kindness shows a positive association with well-being (Otake et al., 2006; Lyubomirsky and Layous, 2013), self-worth (Fredrickson et al., 2008), positive affect, experienced social support, and mindfulness, and a negative association with negative affect and symptoms of illness (Fredrickson et al., 2008; Cohn and Fredrickson, 2010; Hoffman et al., 2011).

#### Three Good Things

The Three Good Things intervention involves writing down three things that went well each day while consciously focusing attention on positive emotions of the day (Seligman et al., 2005). Implementation of the intervention showed a positive association with experiencing positive emotions (Lyubomirsky, 2008), positive affect, gratitude (Martínez-Martí et al., 2010), and well-being, and a negative association with depressive symptomatology (Seligman et al., 2005) and burnout (Adair et al., 2020).

## Associations of 6-Minute Diary Interventions With Psychological Variables

### Stress

According to Lazarus and Folkman’s (1984) transactional stress model, stressful situations are defined as cognitive and emotional appraisal processes of a situation and the coping options available to the person. Thus, if a person feels stressed is dependent on an individual evaluation of the potentially stressful situation and whether the individual feels capable of coping with it.

#### Effects of Stress

How individuals perceive and cope with stress varies from person to person; however, short- to medium-term stress often results in physiological activation, impairment of well-being, and reduced performance (Kaufmann et al., 1982). In contrast to medium- to long-term stress exposure, this is not directly detrimental to health (Werdecker and Esch, 2019). Consequences of prolonged stress include elevated blood pressure, cardiovascular disease, or psychological disorders, such as depression or anxiety disorders (Klein et al., 2016; O’Connor et al., 2021). In addition, perceived stress is associated with lower life satisfaction (Matheny et al., 2008).

#### Preventive Strategies Against Stress

Therefore, in order to avoid or reduce health-damaging consequences of stress, the development of preventive measures is of particular importance. Behavioral prevention strategies focus on enabling individuals to deal with stressful situations. This can be done by expanding existing resources or developing new coping mechanisms. According to findings in the literature, positive emotions (Folkman, 2008; Layous et al., 2014), gratitude, and confidence in particular (Seligman, 2012), facilitate coping with stress.

Furthermore, increasing resilience and self-efficacy expectations can contribute to better coping with stress (Wiley et al., 2017; Werdecker and Esch, 2019). With the Gratitude Intervention, Three Good Things, and Self-Affirmation exercises, the 6-Minute Diary includes several evidence-based brief interventions that could be effective as behavioral prevention against perceived stress.

### Positive and Negative Affect

Positive affect refers to the extent to which a person experiences positive emotional states such as joy, interest, confidence, and alertness. In contrast, negative affect refers to the extent to which a person experiences negative emotional states, such as fear, sadness, anger, guilt, contempt, and disgust (Snyder and Lopez, 2002). Positive and negative affect are defined as dimensions that represent the extent of positive and negative activation. In addition, they influence the extent to which individuals experience life events as joyful and stressful, respectively (Watson and Tellegen, 1999). This means, for example, that individuals with higher positive and lower negative affect will show a more positive emotional response to a highly stressful event than individuals with lower positive and higher negative affect.

#### Effects of Positive and Negative Affect

There are correlations of positive affect with resilience, social activity, satisfaction, and the number of pleasant events, and correlations of negative affect with stress, health problems, and the number of unpleasant events (Watson and Tellegen, 1985; Watson, 1988; Montero-Marin et al., 2015). Fredrickson’s (2001) broaden-and-build theory suggests that positive emotions lead to an expanded repertoire of thinking and action alternatives, which in turn promote the building of resources relevant to success. Experimental evidence suggests that induced positive emotions, compared to neutral and negative states, increase the range of attention (Rowe et al., 2007) and thereby the possibility for more perceived subjective well-being (Fredrickson and Joiner, 2002).

#### Strategies to Increase Positive Affect and to Reduce Negative Affect

Experimental research showed that gratitude interventions (McCullough et al., 2002), goal setting (McCarthy et al., 2010), remembering random acts of kindness (Fredrickson et al., 2008), and the Three Good Things intervention (Martínez-Martí et al., 2010) were associated with increased positive affect. This is supported by a recent review of the effect of positive psychology intervention on positive emotions (Moskowitz et al., 2021). For this reason, the use of the 6-Minute diary could also involve an increase in positive affect. Negative correlations have already been found between the intensity of negative affect and participation in a gratitude intervention (Emmons and Diener, 1986) and a Random Acts of Kindness intervention (Hoffman et al., 2011). For this reason, it is reasonable to speculate that the 6-Minute diary may also lead to a reduction in negative affect.

### Self-Efficacy

General self-efficacy describes a person’s subjective belief that a person can cope with a difficult demand due to their own actions and abilities (Bandura, 1993). Individuals with high self-efficacy consciously set challenging goals, have a high level of motivation, and use their personal strengths to achieve their goals (Luthans et al., 2007).

#### Self-Efficacy as a Resource

A positive attitude of expectation and the subjective conviction to overcome future demands due to one’s own abilities makes self-efficacy a resource in coping with stress (Bandura and Locke, 2003; Schönfeld et al., 2016). Stressful situations produce less subjective stress in individuals with high self-efficacy than in individuals with low self-efficacy (Flammer, 2015). Higher levels of self-efficacy may be protective against post-traumatic stress (Gallagher et al., 2020) and are associated with lower social anxiety and depressive symptoms (Singh and Bussey, 2011). Furthermore, there are positive associations with increased cognitive performance, motivation (Niemiec and Lachowicz-Tabaczek, 2015), overall life satisfaction, subjective well-being, and optimism (Luszczynska et al., 2005; Sica et al., 2015; Schönfeld et al., 2016).

#### Strategies to Increase Self-Efficacy

Self-efficacy is closer to a state than a trait on the spectrum between state and trait and thus changeable within reasonable time and smaller interventions, i.e., can be learned and trained (Luthans et al., 2007; Schönfeld et al., 2016). Self-efficacy is mainly influenced by experiences from four domains (e.g., Bandura, 2000); accordingly, strategies to increase it can also be divided into these domains: (1) Experiences of success: successful attempts in performing certain activities; (2) Model learning: observing successful people who are similar to oneself; (3) Verbal persuasion: other people’s assessment of one’s own ability; and (4) Interpretation of physiological and emotional states: reinterpreting physical states (e.g., trembling, blushing, clammy hands) that are often perceived as signs of failure.

There are several exercises in the 6-Minute diary that are useful for building self-efficacy. Formulating positive goals (“*What would make today wonderful?*” and “*What will I do better tomorrow?*”) leads individuals to take on new challenges, providing opportunities to experience a sense of accomplishment. The Three Good Things intervention allows for reflection on those experiences of success and has already been used as an effective means of increasing self-efficacy (Guo et al., 2020).

### Resilience

Luthans (2002) defines resilience as the ability to bounce back from adversity, conflict, failure, or even positive events, progress, and increasing responsibility and not let them get you down. Thus, resilience describes a functional process of adaptation to adverse or challenging circumstances (Fletcher and Sarkar, 2013; Chadwick, 2014; Davidson et al., 2016; Chmitorz et al., 2018). In contrast, contemporary definitions instead assume that resilience is a trait that can be learned and trained (Luthans et al., 2007; Smith et al., 2016).

#### Impact of Resilience

Empirical research has demonstrated that resilience is closely related to well-being (Ryff, 1989; Keyes et al., 2002; Keyes and Grzywacz, 2005). Evidence suggests that resilience has an impact on positive affect, physical health, sense of purpose, optimism, life satisfaction, and mindfulness (Montross et al., 2006; Lee et al., 2008; Smith et al., 2008; Abolghasemi and Varaniyab, 2010; Huppert and So, 2013; Keye and Pidgeon, 2013). Furthermore, evidence shows a moderating effect of resilience on depressive symptom severity, as well as negative correlations with depression, negative affect, pessimism, and emotion blindness (Smith et al., 2008; Wingo et al., 2010).

#### Strategies to Increase Resilience

Strategies to increase resilience can be divided into three groups with different emphases: risk-, resource-, and process-oriented (Masten et al., 2009), with the former being mainly relevant in a developmental psychology context (Luthans et al., 2007). Resource-oriented strategies focus on building actual or perceived resources. These include increasing self-efficacy expectations, optimism (Luthans et al., 2007), and positive affect (Cohn et al., 2009). Masten et al. (2009) define process-oriented strategies as effective adaptive systems and processes that are activated to appropriately deal with potential risk factors. Strategies from this domain often include elements of self-reflection and self-regulation.

The Gratitude Intervention, Random Acts of Kindness, and Three Good Things exercises from the 6-Minute Diary may lead to increases in positive affect (e.g., Martínez-Martí et al., 2010) and optimism (e.g., McCullough et al., 2002; Watkins et al., 2003), potentially involving increases in resilience (Gallagher et al., 2020). Furthermore, the use of the 6-Minute diary could lead to the development of self-efficacy expectations, which in turn potentially could have a positive impact on the development of resilience.

### Aim of the Study and Hypotheses

The aim of the present study was to investigate the effect of the 6-Minute diary. Building on the above relations, we hypothesized the following:

1. Participants in the intervention diary group will report a greater decrease in perceived stress after (a) 2 (t2) and (b) 4 weeks (t3) than participants in the control group.
2. Participants in the intervention group report a greater decrease in negative affect after (a) 2 (t2) and (b) 4 weeks (t3) than participants in the control group.
3. Participants in the intervention group condition report a greater increase in positive affect after (a) 2 (t2) and (b) 4 weeks (t3) than participants in the control group.
4. Participants in the intervention group condition report a greater increase in resilience after (a) 2 (t2) and (b) 4 weeks (t3) than participants in the control group.
5. Participants in the intervention group report a stronger increase in self-efficacy after (a) 2 (t2) and (b) 4 weeks (t3) than participants in the control group.

## Materials and Methods

We report every outcome variable we assessed in this section. No other dependent variables have been included or excluded in the process of this study.

### Participants

Participants were recruited *via* advertisements in university social networks and had to be at least 18 years old. Participation in the study was voluntary. Students could earn test subject hours for participation. To determine an approximation of required sample size, we calculated an *a priori* power analysis using G*Power (Faul et al., 2007) based on the expected effect [*f* = 0.2, beta = 0.80, α = 0.05; multivariate analysis of variance (MANOVA), repeated measures, between factors] on the outcome measures. The calculation indicated that with a sample size of 80 individuals per group, we would be able to detect the expected effects. We planned to start the intervention with approximately 100 participants to account for a 20% dropout rate.

**FIGURE 1:**
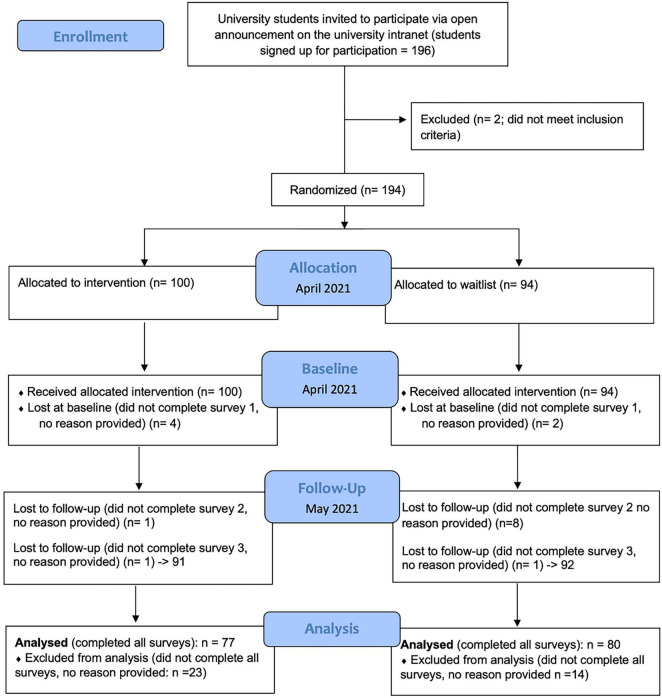
Flow chart – timeline and procedure of current study.

Participants were on average 23.95 (SD = 6.25) years old, and 75.15% were women. Two participants who identified as non-binary were excluded from the analyses due to low numbers, as an *n* of two is not sufficient for any tests of statistical significance. The final sample consisted of *N* = 157 participants, 77 in the intervention group and 80 in the control group (see Figure 1, CONSORT diagram for further details). We ran a dropout analysis to check for differences in variables between drop-outs and participants who completed the final questionnaire. None of the dependent variables, age or gender showed any statistically significant differences between the two groups.

### Intervention

Participants in the intervention group were asked to write two diary entries daily. The diary divided the day into two sections. In the first section, participants were asked to (a) name three things for which they are grateful, (b) describe what would make the current day a good day, and (c) formulate a self-affirmation. In the second section, participants are asked to note (d) what good things they did for others that day, (e) what they will do better the next day, and (f) what Three Good Things they experienced that day.

All participants were informed that they did not have to share their diary entries and that they would not be read by anyone. Furthermore, participants were asked to follow the instructions in the 6-Minute diary and not to write more than one diary entry per day. The control group was designed as a waiting group, so participants did not have to do anything in this condition.

### Procedure

Participants were informed in advance that the study was an investigation of the effectiveness of positive psychology interventions. After enrolling in the study, participants were randomly assigned to either the intervention group or control group. Participants in the 6-Minute diary group were then mailed an intervention packet that included the diary and detailed instructions on how to complete it.

All participants were sent a link to an online survey at the first measurement time point (t1) to collect demographic data, as well as baseline values of all outcome measures. After completion of the first survey, participants in the intervention group were asked to begin completing the diary; participants in the control group were told that additional baseline data would be collected before they could begin the intervention. Two (t2) and 4 weeks (t3) after the first survey, participants in both groups received an email with a link to another online survey. All participants received an e-mail 1 week later with a reminder to complete the questionnaire.

### Instruments

#### Perceived Stress Scale

The German version of the Perceived Stress Scale (PSS-10, Schneider et al., 2020) was used to assess perceived stress. The scale captures to which extent individuals perceive situations in their lives as excessively uncontrollable, unpredictable, and stressful relative to their subjective coping abilities. The 10 items (e.g., “*In the past month, how often have you felt unable to control the important things in your life?*”) are answered on a 5-point rating scale. The scale ranges from 1 = “*never*” to 5 = “*very often*.” Here, higher scores indicate higher levels of perceived stress. Examination of internal consistency across the three measurement time points showed Cronbach’s α between 0.89 and 0.91 and McDonald’s ω between 0.92 and 0.93.

#### Positive and Negative Affect Schedule

Positive and negative affect was assessed using the German version of the Positive and Negative Affect Schedule (PANAS; Krohne et al., 1996). Participants were asked to rate 20 items, in terms of different sensations and feelings, on a 5-point rating scale regarding their intensity over the past 2 weeks. The scale ranges from 1 = “*not at all*” to 5 = “*extremely*,” with 10 adjectives each capturing the dimensions of positive affect (e.g., “*active*”) and negative affect (e.g., “*distressed*”). Higher scores indicate higher levels of positive and negative affect, respectively. Examination of internal consistency across the three measurement time points showed Cronbach’s α ranging from 0.91 to 0.93 for positive affect and 0.82 to 0.88 for negative affect. McDonald’s ω between 0.93 and 0.95 for positive affect and 0.88 and 0.91 for negative affect.

#### General Self-Efficacy Scale

Schwarzer and Jerusalem (2010) General Self-Efficacy Scale was used to assess self-efficacy. Ten items (e.g., “*I face difficulties calmly because I can always trust my abilities*.”) are rated on a 6-point rating scale ranging from 1 = “*Strongly disagree*” to 6 = “*Strongly agree*.” Here, higher scores indicate higher levels of self-efficacy. Examination of internal consistency across the three measurement time points showed Cronbach’s α between 0.91 and 0.92 and McDonald’s ω between 0.92 and 0.94.

#### Brief Resilience Scale

Resilience was assessed using the German version of the Brief Resilience Scale (BRS; Chmitorz et al., 2018). This is based on the resilience definition that resilient individuals have the ability to not succumb to and recover from stress (Smith et al., 2008). Six items (e.g., “*I tend to recover quickly after difficult times*.”) are answered on a 5-point rating scale, with response options ranging from 1 = “*Strongly disagree*” to 5 = “*Strongly agree*.” Here, higher scores indicate higher levels of resilience. Examination of internal consistency across the three measurement time points showed Cronbach’s α between 0.80 and 0.89 and McDonald’s ω between 0.87 and 0.94.

#### Intervention Check

Following Proyer et al. (2015), we asked participants in the intervention group to rate how they liked the intervention using single-item ratings (1 = “*not at all*” to 7 “*very much*”) and to provide a subjective assessment of whether they derived personal benefit from the intervention and, if so, how they quantified that benefit (1 = “*no, not at all*” to 5 = “*yes, very much*”). Furthermore, we asked about compliance and asked participants to rate how much they adhered to the instructions (0% = “*not at all*” to 100% = “*completely*”).

### Data Analysis

#### Preliminary Analysis

Prior to the main analysis, a MANOVA was conducted to examine whether participants in the intervention group and the control group differed in their baseline scores on perceived stress, resilience, positive and negative affect, self-efficacy, gender, and age. Results showed no difference between groups, Pillai’s trace = 0.018, *F*(5,151) = 0.567, *p* = 0.726 (Grissom and Kim, 2012). The Pearson Chi-square test showed no gender differences in the distribution of participants in the conditions [χ^2^(1, *N* = 157) = 0.002, *p* = 0.962, Cramer’s φ = 0.004]. Furthermore, there were no differences in the age structure of the different groups [*t*(155) = −0.972, *p* = 0.33]. Means, standard deviations, and Cronbach’s α for both groups at all three measurement time points are presented in Table 1 on the next page. Preliminary analyses and descriptive statistics were performed using IBM SPSS version 25.

**TABLE 1:** Means, standard deviations, Cronbach’s alpha, and McDonalds omega in the 6-minute diary group and the control group at all three measurement points.

	6-Minute Diary			Control group				

Instrument	*M*	SD	α	ω	*M*	SD	α	ω
**Pretest**								
PSS-10	2.94	0.68	0.91	0.93	2.94	0.75	0.90	0.93
NA	2.26	0.64	0.84	0.89	2.35	0.73	0.87	0.91
PA	3.07	0.75	0.91	0.93	3.01	0.81	0.92	0.95
BRS-6	3.72	0.81	0.89	0.94	3.78	0.92	0.87	0.93
SWE	4.12	0.63	0.91	0.93	4.10	0.75	0.92	0.94
**T2**								
PSS-10	2.62	0.69	0.90	0.92	2.90	0.77	0.91	0.93
NA	2.05	0.68	0.86	0.91	2.29	0.76	0.88	0.91
PA	3.28	0.75	0.91	0.93	3.12	0.81	0.93	0.95
BRS-6	3.85	0.93	0.80	0.87	3.77	0.89	0.85	0.92
SWE	4.30	0.69	0.91	0.94	4.19	0.77	0.92	0.94
**T3**								
PSS-10	2.49	0.66	0.89	0.92	2.80	0.74	0.90	0.93
NA	1.93	0.60	0.82	0.88	2.23	0.74	0.86	0.90
PA	3.40	0.71	0.91	0.93	3.76	0.80	0.92	0.94
BRS-6	4.05	0.79	0.83	0.90	3.76	0.95	0.88	0.92
SWE	4.39	0.67	0.91	0.92	4.17	0.77	0.91	0.93

*PSS-10, perceived stress; NA, negative affect; PA, positive affect; BRS-6, resilience; SWE, self-efficacy expectancy; M, mean; SD, standard deviation.*

#### Main Analysis

**FIGURE 2:**
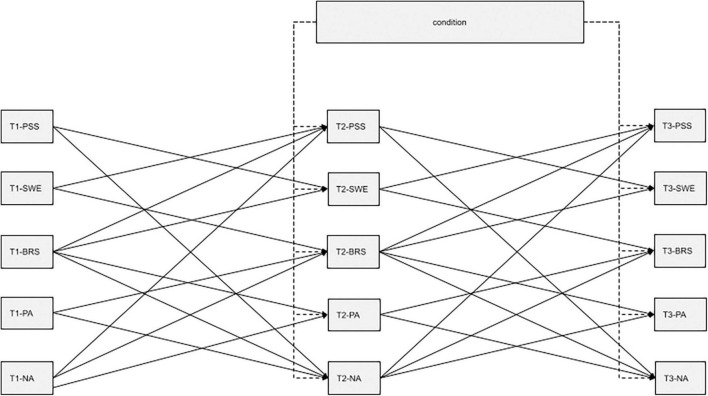
Conceptual – cross-lagged effects model over all measurement points.

We tested the hypotheses using path analyses. For this, we specified a structural model with autoregressive and cross-lagged effects to control for correlations within measurement time points and autoregressive influences (Kearney, 2017). Variables were allowed to correlate within their respective measurement time points. We specified seven theoretically significant cross-lagged effects: negative affect and (1) positive affect, (2) stress, and (3) resilience; (4) stress and self-efficacy; and resilience and (5) positive affect, (6) self-efficacy, and (7) stress. An overview of all the cross-lagged effects can be seen in Figure 2. We used Hu and Bentler’s (1999) standard criteria to evaluate the structural model. The results indicate that the specified model represents the data well [χ^2^(37) = 18.69, *p* = 0.995, SRMR = 0.023, RMSEA = 0.000, CFI = 1.00, TLI = 1.031]. The robust maximum likelihood estimator was used as the estimation method for the path analysis to correct for the non-normal distribution of the data. For path analysis, we used the package “lavaan” (Rosseel, 2012) of the statistical software R (version 4.0.3).

## Results

Our first hypothesis stated that participants in the intervention group would report a greater decrease in perceived stress after 2 (hypothesis 1a) and 4 weeks (hypothesis 1b) than participants in the control group. The results of the regression analysis (Table 2) show that participants in the intervention group report statistically significantly less perceived stress than participants in the control group after 2 weeks (*B* = −1.234, *p* = 0.002), but not after 4 weeks (*B* = −0.649, *p* = 0.088). Thus, the results only provide evidence in favor of hypothesis 1a; hypothesis 1b is accordingly rejected. According to our second hypothesis, participants in the intervention group report a greater decrease in negative affect after 2 (hypothesis 2a) and 4 weeks (hypothesis 2b) than participants in the control group. Results indicate that participants in the intervention group report statistically significantly less negative affect at 2 (*B* = −0.945, *p* = 0.023) and 4 weeks (*B* = −0.782, *p* = 0.031), providing evidence in favor of hypotheses 2a and 2b. The third hypothesis related to the relative increase in positive affect for participants in the intervention group compared to the control group at both time points (t2: hypothesis 3a, t3: hypothesis 3b). The regression coefficients show no statistically significant effects, i.e., there are no differences in the increase of positive affect between the two conditions. For this reason, hypothesis 3 is rejected. According to our fourth hypothesis, participants in the intervention group report a greater increase in resilience after 2 (hypothesis 4a) and 4 weeks (hypothesis 4b) than participants in the control group. We also found only limited evidence for this set of hypotheses. The increase in resilience after 2 weeks did not differ statistically significantly across conditions. However, it appears that participants in the intervention group reported statistically significant higher resilience scores than the control group after 4 weeks (*B* = 0.763, *p* = 0.005). A similar picture emerges for the fifth hypothesis, which states that participants in the intervention group report a greater increase in self-efficacy after 2 (hypothesis 5a) and 4 weeks (hypothesis 5b) than participants in the control group. Again, the increase in self-efficacy is not statistically significant after 2 weeks, but is significant after 4 weeks (*B* = 0.574, *p* = 0.043). These results provide evidence only in favor of hypothesis 5b (for the results of all regressions, see Table 3).

**TABLE 2:** Unstandardized and standardized coefficients, standard errors, and *p*-values of the regressions.

Regression	*B*	SE *B*	*p*	β
Stress t2 on condition	–1.234	0.396	**0.002**	–0.170
Stress t3 on condition	–0.649	0.380	0.088	–0.092
Negative affect t2 on condition	–0.945	0.415	**0.023**	–0.132
Negative affect t3 on condition	–0.782	0.362	**0.031**	–0.120
Positive affect t2 on condition	0.483	0.403	0.231	0.065
Positive affect t3 on condition	0.702	0.370	0.057	0.095
Resilience t2 on condition	0.007	0.268	0.978	0.001
Resilience t3 on condition	0.763	0.275	**0.005**	0.136
Self-efficacy t2 on condition	0.421	0.318	0.186	0.068
Self-efficacy t3 on condition	0.574	0.283	**0.043**	0.092

*Coefficients estimated with robust maximum likelihood method; t2, second time point after 2 weeks; t3, third time point after 4 weeks; bold values indicate statistical significance; SE, standard error.*

**TABLE 3:** Unstandardized and standardized coefficients, standard errors, and *p*-values of the cross-shifted regressions.

Regression	*B*	SE *B*	*p*	β
Stress t3 on negative affect t2	0.139	0.064	**0.030**	0.140
Stress t2 on negative affect t1	0.338	0.061	**<0.001**	0.306
Stress t3 on resilience t2	0.091	0.097	0.348	0.063
Stress t2 on resilience t1	0.087	0.094	0.355	0.061
Stress t3 on self-efficacy expectation t2	–0.117	0.057	**0.039**	–0.102
Stress t2 on self-efficacy expectation t1	–0.133	0.069	0.053	–0.114
Negative affect t3 on positive affect t2	0.006	0.051	0.899	0.007
Negative affect t2 on positive affect t1	0.121	0.062	**0.050**	0.128
Negative affect t3 on resilience t2	0.029	0.097	0.766	0.022
Negative affect t2 on resilience t1	–0.123	0.085	0.149	–0.088
Negative affect t3 on stress t2	0.015	0.093	0.871	0.017
Negative affect t2 on stress t1	0.153	0.106	0.148	0.150
Positive affect t3 on negative affect t2	–0.079	0.072	0.273	–0.077
Positive affect t2 on negative affect t1	–0.133	0.062	**0.033**	–0.188
Positive affect t3 on resilience t2	–0.013	0.100	0.896	–0.009
Positive affect t2 on resilience t1	0.015	0.092	0.875	0.010
Resilience t3 on negative affect t2	–0.007	0.064	0.918	–0.008
Resilience t2 on negative affect t1	–0.024	0.083	0.773	–0.032
Resilience t3 on positive affect t2	0.078	0.061	0.199	0.104
Resilience t2 on positive affect t1	–0.018	0.040	0.656	–0.009
Resilience t3 on stress t2	0.092	0.115	0.421	0.117
Resilience t2 on stress t1	–0.229	0.137	-0.229	–0.336
Resilience t3 on self-efficacy expectation t2	0.113	0.076	0.136	0.124
Resilience t2 on self-efficacy expectation t1	0.049	0.067	0.459	0.061
Self-efficacy expectation t3 on resilience t2	0.045	0.078	0.561	0.036
Self-efficacy expectation t2 on resilience t1	0.123	0.076	0.105	0.102
Self-efficacy expectation t3 on stress t2	–0.079	0.056	0.159	–0.92
Self-efficacy expectation t2 on stress t1	–0.159	0.054	**0.003**	–0.181

*Coefficients estimated with robust maximum likelihood method; t1, pretest; t2, second time point after 2 weeks; t3, third time point after 4 weeks; bold values indicate statistical significance; SE, standard error.*

Regarding the intervention checks, participants in the intervention group reported that they enjoyed the exercise (Mt2 = 5.48, SD = 1.21; Mt3 = 5.17, SD = 1.40) and benefited from doing it (Mt2 = 3.61, SD = 0.95; Mt3 = 3.53, SD = 0.93). Subjects also reported high scores on compliance (Mt2 = 84.48, SD = 12.67; Mt3 = 79.94, SD = 16.69). The means of all three measures decrease across the measurement time points, so we used *t*-tests to test whether this decrease was statistically significant. In the case of intervention preference [*t*(76) = 2.625, *p* = 0.010], as well as compliance [*t*(76) = 7.094, *p* = 0.001], there was a statistically significant reduction. In contrast, the decrease in subjective perceived benefit of the intervention was not statistically significant, *t*(76) = 0.847, *p* = 0.400.

## Discussion

The present study evaluated the effectiveness of a combination of positive-psychology interventions included in the commercial 6-Minute diary in a student population compared with a wait-list control group. Results provide evidence that the intervention positively influenced four of the five outcome measures (i.e., perceived stress, resilience, negative affect, and self-efficacy). Only the positive affect was not statistically significantly influenced by the intervention compared to the control group in the current study.

The decrease in perceived stress at the second time point suggests that the interventions had a rapid effect on perceived stress. However, at the third measurement time point, perceived stress did not decrease further to a statistically significant level, which could indicate that the interventions buffer stress but cannot completely negate it. This is also supported by the fact that the best predictor of perceived stress is the autoregressive effect of stress from the previous time point.

The data showed a statistically significant increase in self-efficacy and resilience scores only after 4 weeks, suggesting that change in these constructs takes time. These results are consistent with the assumption that states and personality traits exist on a continuum and that certain state variables (e.g., self-efficacy) also exhibit some characteristics of personality dispositions and vice versa (Luthans et al., 2015). States are volatile and easily changeable, whereas dispositions are considered stable and difficult to change. In addition to self-efficacy, resilience also tends to be state-like (rather than a pure state variable) on the continuum, which may be one reason for the delay in change.

Regarding affect, the study found that the use of the 6-minute diary resulted in lower levels of negative affect at the second and third measurement time points, while positive affect remained unchanged. One possible explanation for this would be that the interventions tended to provide a buffer against negative affect – similar to the perceived stress mentioned above – but did not increase positive affect *per se*. Another possible explanation for the lack of change in positive affect could be found in the interaction with resilience. Smith et al. (2016) found that individuals with higher resilience reported higher levels of positive affect. Future studies could examine, for example, whether changes in positive affect are mediated by resilience can be mapped over longer periods of time. The investigation of longer longitudinal intervals could also be useful in order to shed more light on the possible decrease of compliance, and at the same time interventions to stop this decrease should be investigated.

### Practical Implications

The intervention checks showed that participants in the intervention group reported high scores on preference, compliance, and utility at both, the second and third, measurement time points. Preference and compliance decrease somewhat over time, but still remain at high levels. Perceived usefulness remains consistently in a high range of values, indicating that participants subjectively benefit from performing the exercises in the 6-Minute diary. In order to prevent a further decline in the participants’ compliance, the use of external reminders could be worthwhile. This could possibly stabilize participants’ high level of compliance long enough for a habitualization effect to develop.

Through the use of the 6 Minutes Diary, the interventions are made available for a wider audience that otherwise might not have gotten in touch with these interventions, allowing for more people to benefit from the exercises. The 6-Minute Diary retails in Germany at approximately 25€ and provides pages for 6 months. It is thus relatively low in both costs and time commitment. Nevertheless, it might not be financially accessible for everyone. On the other hand, individuals could do the interventions *via* pen and paper or the note app on a smartphone, thereby not having to spend any extra money. However, it is possible that due to the financial investment, individuals feel a higher commitment to actually using the diary (Rusbult et al., 2011). Also, the design of the diary is visually appealing and might aid in keeping the motivation for doing the exercises high, even after the novelty effect wears off. Future studies could compare the effects of the intervention on individuals using the 6-Minute Diary (or other commercial versions) with individuals using pen and paper. Of special interest could be the change in compliance and preference for either of the conditions. Of particular interest here might be research that looks at which individuals have higher compliance with commercially available products and designs of interventions. In this way, interpersonal differences could be identified in order to optimize the fit between person, intervention and presentation in order to increase possible effects (e.g., Nelson and Lyubomirsky, 2014; Sheldon and Lyubomirsky, 2021).

### Limitations

Discussing specific results of the study, there are possible limitations to keep in mind. First, our sample consisted of German students, most of them women. Previous evidence suggests that the effects of positive-psychology interventions vary between different populations (Bolier et al., 2013), it is possible that our results cannot be generalized to other non-student populations. Since our study only examined students, future work should also examine other populations. Second, we only used positive self-report measures that are prone to possible biases, e.g., the issue of common method variance. Even though there is an ongoing debate about the extent of the potential problem (e.g., Pace, 2010; Cooper et al., 2020), it should be kept in mind. Third, we used manifest variables in our statistical analysis and thus our model could possibly underestimate the stability of the used constructs over time (Eid et al., 2017). The use of manifest variables was necessary because the sample for a model that includes measurement and structural model would have significantly exceeded the number of participants in our intervention study. Forth, the use of a waitlist control condition is possibly a limitation of the present study. Placebo effects cannot be completely ruled out and meta-analyses showed that the type of comparison sample could affect effectiveness estimates (e.g., Sin and Lyubomirsky, 2009).

## Conclusion

Our results provide first evidence for the positive influence of the 6-minute diary. However, some points should be considered in future research. Since stress has been found to be a time-sensitive construct, more frequent shorter time intervals between data collection periods should be considered. This would allow for a closer look at the impact of the diary intervention on stress. The delayed significant increase in resilience and self-efficacy after 4 weeks, on the other hand, calls for future studies over a longer period of time. This could allow conclusions to be drawn as to whether and to what extent dispositions can also be influenced by the intervention.

The results of our study indicate a positive influence of the 6-minute diary. In particular, this self-intervention seems to have a positive effect on self-efficacy and resilience. Analogous to resilience, our results suggest a protective function of the intervention to reduce negative affect. In summary, the 6-minute diary seems to be a helpful tool for building positive resources that requires relatively little financial investment and is easily integrated into one’s life.

## Data Availability Statement

The original contributions presented in the study are included in the article/Supplementary Material, further inquiries can be directed to the corresponding author.

## Ethics Statement

Ethical review and approval was not required for the study on human participants in accordance with the local legislation and institutional requirements. The patients/participants provided their written informed consent to participate in this study.

## Author Contributions

TL and MA contributed to conception and design of the study. MA and BB organized the database and performed the statistical analysis. TL, MA, and BB wrote sections of the manuscript. All authors contributed to manuscript revision, read, and approved the submitted version.

## Conflict of Interest

The authors received the intervention material, as well as a student assistant for the organization of distribution of the material from the organization UrBestself. UrBestself had no influence on the experimental design, statistical analysis, or the manuscript.

## Publisher’s Note

All claims expressed in this article are solely those of the authors and do not necessarily represent those of their affiliated organizations, or those of the publisher, the editors and the reviewers. Any product that may be evaluated in this article, or claim that may be made by its manufacturer, is not guaranteed or endorsed by the publisher.
